# Early in life effects and heredity: reconciling neo-Darwinism with neo-Lamarckism under the banner of the inclusive evolutionary synthesis

**DOI:** 10.1098/rstb.2018.0113

**Published:** 2019-02-25

**Authors:** Étienne Danchin, Arnaud Pocheville, Philippe Huneman

**Affiliations:** 1 Laboratoire Évolution and Diversité Biologique (EDB UMR 5174), Université de Toulouse Midi-Pyrénées, CNRS, IRD, UPS, 118 route de Narbonne, Bat 4R1, 31062 Toulouse cedex 9, France; 2 Department of Philosophy and Charles Perkins Centre, University of Sydney, Sydney, New South Wales 2006, Australia; 3 Institut d'Histoire et de Philosophie des Sciences et des Techniques, CNRS/Université Paris I Panthéon-Sorbonne, Paris, France

**Keywords:** epigenetics, timescales, Weismann barrier, modern synthesis, inclusive evolutionary synthesis

## Abstract

Recent discoveries show that early in life effects often have long-lasting influences, sometimes even spanning several generations. Such intergenerational effects of early life events appear not easily reconcilable with strict genetic inheritance. However, an integrative evolutionary medicine of early life effects needs a sound view of inheritance in development and evolution. Here, we show how to articulate the gene-centred and non-gene-centred visions of inheritance. We first recall the coexistence of two gene concepts in scientific discussions, a statistical one (focused on patterns of parent–offspring resemblance, and implicitly including non-DNA-sequence-based resemblance), and a molecular one (based on the DNA sequence). We then show how all the different mechanisms of inheritance recently discovered can be integrated into an inclusive theory of evolution where different mechanisms would enable adaptation to changing environments at different timescales. One surprising consequence of this integrative vision of inheritance is that early in life effects start much earlier than fertilization.

This article is part of the theme issue ‘Developing differences: early-life effects and evolutionary medicine’.

## 1. Introduction

The current mainstream view of inheritance in evolution is that the only real source of heredity lies in the DNA sequence, in particular in the germline that is considered fully isolated from the rest of the organism and its environment, ‘sealed off from the outside world’ [1]. In this genocentric view of inheritance, early in life environmental effects can strongly affect an organism for its whole lifespan, but such effects cannot percolate into the next generation. The fantastic technological developments of molecular biology since the 1990s, although entirely placed within this view, eventually led to the finding that the accounting of the sole genetic variation does not explain inheritance in all its complexity [2]. That finding triggered major debates, notably about missing heritability, the fact that estimates of heritability in population genetics or epidemiology are almost always much higher than those obtained in genome-wide association studies [3–7]. The ubiquity of this discrepancy on its own suggests that a genes-only view of heredity may be far from sufficient to explain trait inheritance.

In parallel, ever since the early days of neo-Darwinism and even more so since the mid-2000s [3,8–10], evidence mainly coming from molecular studies accrued that plastic adaptive responses sometimes become ‘inclusively heritable’ ([11–13], reviews in [14–19]). In particular, recent discoveries of exquisite molecular mechanisms of inherited early in life effects show that early life effects often have long-lasting influences, sometimes even spanning several if not many generations. Such intergenerational effects of early life challenges are not easily reconcilable with strict genetic inheritance, and raise many, sometimes provocative, questions such as ‘when does early life start?’ or ‘is there a somewhat Lamarckian component to acknowledge in evolution?’ Such questions have fuelled a vivid and more and more audible debate about the necessity to revise the Modern Synthesis of evolution, the current frame of evolutionary theory, in order to incorporate the patterns of inheritance that cannot be explained by conventional mechanisms [8,20,21].

We show here how the recent fascinating discoveries that in particular integrate early in life effects have some Lamarckian flavour and how integrating them into the modern synthesis of evolution has the potential to reconcile our neo-Darwinism (the current Modern Synthesis) with the Lamarckian element into a single inclusive evolutionary synthesis, which closely resembles the Darwinism of the origin. We will show that the key element of reconciliation lies in the fact that all processes of parent–offspring resemblance seem to act and enable adaptation at very different timescales and thus do not conflict with but rather complement each other. A central aspect of the current research programme calling for an extended [20,22,23] or inclusive [5,15,24] synthesis is that all examples of transmission of acquired adaptations largely lead us to reconsider concepts of heredity and call for the crystallization of a much broader concept of inheritance [19].

We first briefly describe the history of the gene concept, and define our main concepts in box 1. We then analyse the different timescales of accommodation and adaptation to re-explore the sources of phenotypic variation. We briefly illustrate recently discovered molecular mechanisms of non-genetic inheritance and highlight their very different timescales and reversibility potentials. This leads us to underline the complementarities of these inheritance systems within a continuum of timescales along which they can be ranked. In doing so, we stress the importance of this emerging approach for medicine in general and for early in life effects in particular. We illustrate how these considerations have the potential to open major avenues to establish new therapies for many of the inherited human disorders, most of which originate in the early part of an individual's life or of its ancestors' life. Finally, we show how this emerging inclusive evolutionary synthesis rejuvenates Darwinism from neo-Darwinism and how the inclusive vision of inheritance that emerges from recent discoveries leads us to accept a neo-Lamarckian component within a Darwinian framework.

Box 1. **Glossary.**

**Accommodation**: The process by which individual organisms respond to environmental change through phenotypic plasticity. It allows organisms to improve their fit to their current environment. It unfolds within one generation.

**Adaptation (by natural selection)**: The process by which natural selection affects inclusively heritable variation across generations in a way that increases the fit of the organisms to their environment.

**Development**: The processes by which environmental factors interact with gene expression in building the phenotype. Here we argue that this process starts before fertilization as epigenetic marks inherited from environmental effects on sometimes ancient ancestors participate in the building of the phenotype.

**Evolution**: The process by which the frequencies of variants (be they genetic or not) change over generations.

**Genetic**: Encoded into the DNA sequence, whether coding or not. All other mechanisms of heredity that do not rest on variation in the DNA sequence constitute non-genetic inheritance.

**Genocentrism**: The gene (i.e. sequence) centred vision of inheritance, and therefore often of medicine or evolution.

**Heredity**: Patterns of parent–offspring resemblance. Today, it is widely accepted in biology that heredity results from parents transmitting information to their offspring, though the deep nature of this information is still at the heart of a hot debate [25–28]. Heredity is the cornerstone of evolution through natural or artificial selection, as well as through drift.

**Heritability**: The part of phenotypic variation that results from genetic variation, either additive (narrow-sense heritability) or total (broad-sense heritability). Constitutes the concept of parent–offspring resemblance at play in quantitative genetics. Although rarely stated it is implicitly supposed to result only from variation in DNA sequence among individuals.

**Heritability (inclusive**): Statistical term quantifying the degree of parent–offspring resemblance, whatever the mechanisms responsible for it (whether genetic or not; [15,29]).

**Inclusive Evolutionary Synthesis**: The extension of the Modern Synthesis that includes all components of inheritance and their interactions. Its ambition is to incorporate any known forms of parent–offspring resemblance, including epigenetic, ecological and cultural inheritance, parental effects of all sorts, as well as the inheritance of microbiota or the effects of any molecular memory system such as, for instance, in prions.

**Inheritance:** Mechanisms that produce heredity. Inheritance involves some forms of information transmission from parents to offspring. We distinguish inheritance (the mechanisms) from heredity (the pattern) [30].

**Inheritance systems**: Categories of mechanisms of parent–offspring resemblance.

**Modern Synthesis of evolution**: The merging of Darwinian approaches studying natural selection with genetics elaborated on the basis of population genetics by Haldane, Fisher, Wright, Mayr, Dozhansky, Simpson, Huxley, Rensch and others in the 1940s and 1950s [31–35].

**Natural selection**: A process occurring when (i) there is phenotypic variation within a population (ii) that is inclusively heritable and (iii) that causally impacts fitness (including survival and reproduction).

**Neo-Darwinism**: Theory posterior to Darwin in which species evolve; natural selection is the major process accounting for evolution; variation is blind; inheritance is of a genetic nature. Thus, it appears as the restriction of Darwinism to a theory of evolution without the inheritance of acquired characteristics [36].

**Non-genetic inheritance**: Mechanisms of heredity that do not rest on variation in the DNA sequence [5,15,29].

**Sequencic**: To avoid the ambiguity of gene concepts and the negative shade of the term genocentrism, one could use the term ‘sequencic’ when reducing genetics to the sole DNA sequence of nucleotides.

## 2. A brief history of the gene concept

Definitions of the gene concept can be split into two categories, which can be traced to the seminal work of Mendel [37] and still coexist within contemporary biology (see [38]). One concept is based on the statistical quantification of parent–offspring resemblance [39]. It quantifies the proportion of phenotypic variation that is transmitted to offspring (i.e. is inclusively heritable), which constitutes the core condition of evolution by artificial or natural selection or drift. This concept persists in quantitative and population genetics, as well as in epidemiology.

The second gene concept is mechanistic: it aims at representing the mechanism responsible for parent–offspring resemblance. Ever since Darwin, this topic has been subject to speculation. Darwin's vision of inheritance (which was anterior to the definition of a gene concept) included a mechanism that was reminiscent of what we now call Lamarckism: the ‘effects of use and disuse’ [40, p. 134]. To him, and some of his successors who were to be called the neo-Lamarckians, inheritance was soft, pliable [41, p. 687]. This vision contrasted with that of the neo-Darwinians, especially the founding fathers of the Modern Synthesis, who drew a sharp line between inheritance and developmental mechanisms. To them, inheritance was hard, unchangeable, except by rare mutations. The soft elements were considered negligible with respect to evolution. With the discovery of the chromosomes [42,43] and, later, of the DNA molecule [44], the gene concept became more and more molecularly tinted, to the point that, now, a gene is commonly thought of as a piece of DNA and genetic information as the information encoded into the DNA sequence (see [45]). The stability of the DNA sequence also seems to justify the concept of hard inheritance at work in evolutionary theory [1].

Philosophers have been questioning the relation of the ‘classical gene concept’ (from Morgan and Castle's genetics), addressed in statistical terms, to the ‘molecular genes’ (DNA sequences), handled by molecular biology, and stressed the conceptual issues of identifying the latter to the former [46], often arguing that we use at least two concepts of the gene (e.g. [2,47–49]). Nevertheless, we easily slip from the purely statistical to the purely DNA-sequence definitions of the gene [38]. For instance, after having shown that such or such trait is heritable, one automatically looks at DNA sequencing, ignoring alternative mechanisms of inheritance. Furthermore, when reading papers from before the 1950s, we now forget that concepts of genetics were then purely statistical (namely, anything that is inherited), and nonetheless interpret those papers in light of our current mainstream ‘sequencic’ vision of genetics.

Here we adopt the sequence definition of the gene (box 1), which, although highly reductionist, has the great merit of being extremely specific, and clearly separating sequence information from other DNA-linked components of inheritance such as epigenetics. Although these two components of inheritance are purely molecular, they have contrasting properties and carry different components of inheritance so that they need to be separated in order to study their respective roles and complementarities [5,15,19,50].

One hit to the DNA-sequence vision of inheritance was the discovery at the turn of the twenty-first century that only a small fraction of DNA on the chromosomes contains coding sequences. The non-coding part of the DNA was first dubbed with the rapidly abandoned term of ‘junk DNA’. The existence of large chunks of non-coding DNA made an easy culprit for the paradox (called the C-value paradox) identified in the 1970s, that the amount of nucleotides in cells does not correlate to organisms' complexity. The sequencing of whole genomes later raised the ‘G-value paradox’: even once we set aside the non-coding DNA, the amount of coding genes did not seem to correlate with organisms' complexity [51]. For example, on average a plant genome contains twice as many genes as an animal genome, which itself contains twice as many genes as a fungal genome [52]. Hence, the amount of coding DNA sequences appears insufficient to account for the development and functioning of complex organisms. To account for transgenerational resemblance, organisms must transmit more than the coding DNA sequences, or at least those sequences should be accompanied by a substantial set of regulatory molecules in states that persist over generations.

Despite the fact that DNA-sequence definitions of the gene now dominate, this pervasive vision has been regularly questioned (e.g. [53]). This questioning gained a lot of momentum since the beginning of the twenty-first century, mainly because the extraordinary successes of the dominant vision also highlighted its limitations. Among others, a challenge came from accruing evidence for the existence of non-genetic (non-DNA-sequence-based) forms of inheritance, to which we turn now.

## 3. The reality of inheritance of acquired environmental effects

Mechanisms of non-genetic inheritance show a high level of molecular sophistication. We provide here and in boxes 2–5 a few examples.

Box 2. **Non-genetic transmission of acquired maternal behaviour.**

In mammals, variation in maternal care can strongly affect reproductive success. In rats, female pups raised by highly caring females (the normal situation) show low levels of methylation of the promoter of genes coding for receptors to sexual hormones, while female pups regularly removed from their mother's care show high levels of methylation of the promoter of these genes [54–57], which leads to the silencing of the corresponding genes. These epigenetic marks being maintained for life, when these female pups become mothers, those that were raised by highly caring females do express those receptor genes in their brain, which makes them sensitive to their own sexual hormones, triggering a cascade of molecular changes that lead them to become fond of their pups and thus to take care of them. In contrast, females that were artificially separated from their mother (mimicking an apparently low-caring mother) do not express their highly methylated sexual hormone receptor genes. They thus cannot sense their own sexual hormones, hence inhibiting the cascade of molecular changes that would have led them to become fond of their pups. As a consequence, they neglect their babies, as their mothers apparently did. As a result, females that were apparently neglected by their mothers become truly neglecting mothers with their own pups. The latter will thus have highly methylated receptor genes in their brain, and will thus become low-caring mothers in their turn. And so on, across many generations.

In this example, the behavioural consequences of the pattern of methylation in mothers become the environmental cause of the establishment of similar epigenetic marks in their developing daughters, and so on over many generations, leading to persistent mother–daughter resemblance in maternal care. A putative adaptive function of the resulting variation in maternal care may be that low maternal care in stressful environments produces offspring that better cope with stress [58] (for a review on such germline-independent inheritance of behaviour, see [59]).

Box 3. **Non-genetic transmission of acquired fear.**

A really surprising example of inherited acquired adaptation concerns the inheritance of acquired fear and the uncovering of part of the molecular pathways underlying such transgenerational effects. For many organisms odour sensitivity constitutes the main sense for fitness-affecting activities such as food finding or predator detection. This is the case for mice that have developed a fantastically fine sense of smell owing to a large family of olfactory receptor genes each involved in the perception of a specific volatile molecule. Mice can decompose even the most complex odour bouquet in a way that would make the best human nose look very primitive. Each olfactory receptor neuron of the nasal epithelium expresses only one of these genes [60]. Based on this specific knowledge about the neurology and genetics of odour reception, carefully designed experiments demonstrated that parent mice conditioned by the association between a benign odour and a mild electric shock transmit their acquired fear to their offspring and grand-offspring through either the male or female gamete [13]. It was shown that the specific gene activated by the specific odour detection was hypomethylated in their gametes. In effect, after *in vitro* fertilizations of unexposed female ova by sperm of exposed males (or vice versa), this methylation pattern was transmitted to unconditioned F1 and F2 offspring that both feared the same odour (but not another) when first exposed to it. *In vitro* fertilization and cross-fostering further showed that this inheritance was not due to any kind of social transmission [13]. It thus appears that an acquired fear of a benign odour can be inherited for at least two generations through epigenetic modifications of the germline. This constitutes another example of the reality of the inheritance of variation that was acquired by recent ancestors (a situation that justifies the extension to the left of the green area in figures 3 and 5).

Box 4. **Non-genetic transmission of environmentally triggered responses.**

One of the first well documented example of inheritance of acquired response to environmental stressors concerns the transgenerational action of environmental toxins such as endocrine-disruptors (methoxychlor and vinclozolin) commonly used in the wine industry through modifications of the male germline [61]. In that study in rats, nearly all F1 to F4 male descendants of F0 pregnant females treated with such chemicals showed strongly decreased fertility concomitant with unusual methylation patterns in the testes. These were transmitted over at least four generations by male but not female gametes despite the fact that only the F0 female received the hormone disruptor [61,62]. Similar multigenerational effects were found with other contaminants [63]. Furthermore, the expression of over 400 genes in F3 appeared affected by the treatment three generations before [64], and preference tests showed that F3 females (but not males) of treated F0 pregnant mothers (as well as females with no history of exposure) preferred males whose ancestors were not exposed to endocrine disruptors over males whose ancestors were exposed three generations before, suggesting that such effects can deeply affect fitness of descendants and thus the course of evolution [65].

Box 5. **Non-genetic transmission of acquired sexual preferences.**

In *Drosophila melanogaster*, a combination of six experiments and modelling showed that female mating preference meets the five criteria of culture and cultural transmission [66]. Fruit fly females (i) express strong social learning in mating preferences, which functions (ii) across age-classes, and is (iii) memorized for sufficient time to be copied. Furthermore, the socially acquired mating preference is (iv) trait-based implying that females learn to prefer males of a given phenotype over males of another phenotype. A fifth experiment showed that fruit fly females show (v) amazingly strong conformity in mate-copying. A model showed that the characteristic measured in the fruit flies may readily lead to the emergence of long-lasting local traditions of preferring a given male phenotype in populations of sizes that can be found in nature. Finally, (vi) the acquired mate preference was maintained in a group of six flies along chains of cultural transmission for much longer than expected by chance in a way that closely match the predictions of our model. These results suggests that the taxonomical range of culture might be much broader than ever envisioned, and that cultural inheritance might have been a significant part of evolutionary processes for extended periods of time. More generally, Kasper *et al.* [67] have argued that, for the inheritance of complex behaviour such as cooperation, it is of prominent importance to integrate the non-genetic component of inheritance.

### (a) Non-genetic transmission of acquired metabolic disorders

One of the most striking examples of inheritance of environmentally triggered early in life responses links parental dietary environment to the phenotype of their descendants. Human metabolic disorders characterizing obesity and diabetes are well known consequences of the diet [11,68,69]. However, recent experiments with mice showed that metabolic disorders acquired before reproduction are transmitted to the offspring via sperm cells. Chen *et al*. [12] triggered metabolic disorders in mice by providing a high-fat diet (HFD). They then realized *in vitro* fertilization, injecting into an oocyte (from a healthy mother) one sperm cell head from a HFD male. The resulting male offspring developed the full metabolic disorder (glucose intolerance and insulin resistance), even if fed with a healthy diet, demonstrating that the sperm head contains all the information for that disorder [12]. Injecting a specific fraction of RNA extracts from the sperm of HFD males into zygotes from normal parents leads the resulting male offspring to develop the glucose intolerance part of the disorder (but not the insulin resistance component), suggesting that part of the inherited information for the development of the disorder is carried out by that small fraction of sperm RNA extracts [12]. Furthermore, these sperm RNAs are plausibly incorporated into sperm cells during their transit through the epididymis [11]. The lumen of the epididymis duct has many RNAs containing micro-vesicles. By fusing with sperm cells, these vesicles probably incorporate their RNA content into the sperm cells in a surprising form of soma-to-germ-communication with transgenerational effects.

This body of research has major potential for medical sciences [68]. Obesity and diabetes linked metabolic disorders constitute a major global public health issue, with an estimated population at risk increasing and approaching one billion people [70]. One may hypothesize that part of the increase is because the acquired diabetes is inclusively heritable, which generates a form of intergenerational snowball effect.

More generally, the parental dietary environment is strongly suspected to affect offspring's health for several generations. In humans, for instance, the ‘thrifty phenotype hypothesis’, elaborated in the 1990s, states that epigenetic programming adapts the fetus to a parental environment in which offspring of dams that experienced food instability during pregnancy develop obesity if they end up in an environment with rich and stable food resources [71]. Thus, ‘paradoxically, rapid improvements in nutrition and other environmental conditions may have damaging effects on the health of those people whose parents and grandparents lived in impoverished conditions’ [72]. For instance, the Dutch Famine that occurred during World War II in parts of the Netherlands generated long-term effects on concerned individuals, but also in their offspring and grand-offspring [73,74], suggesting that such dietary environmental effects on F0 parents can be transmitted at least to F2 offspring. The discovery of the fine molecular mechanisms involved in the development and transmission of this disorder constitutes a striking example of how evolutionary medicine of early in life effects can bring valuable insights opening major potential avenues to define new therapeutic approaches to this major public health issue.

### (b) The contrasting timescales of non-genetic inheritance

Discoveries such as the example above (see also examples in boxes 2–5) challenge the view of inheritance widely accepted within the Modern Synthesis of evolution (review in [14]). For instance, the widely accepted concept of the Weismann barrier between somatic cells and germ cells in animals needs to be revisited as not only is the germline not isolated from the external world, but there are also specific and sophisticated mechanisms that seem to modify the germline in response to environmental change. This conclusion was already suggested by the study of the developmental origin of germ cells and the fact that the determination of which cell produces the germline is also influenced by the effect of surrounding extra embryonic cells (for a review, see [75]). Such mechanisms have a Lamarckian flavour, strongly at odds with the Modern Synthesis' view, which puts ‘blind heritable variation’ as the cornerstone of its concept of evolution by natural selection [76,77]. However, the above examples do not necessarily go against the view of the Modern Synthesis, but may rather complement and broaden its scope as they illustrate additional processes working at very different timescales.

For example, the two examples of non-genetic inheritance, that of maternal behaviour (box 2) and that of fear conditioning (box 3), are likely maintained over a few generations only. Nonetheless, they participate in parent–offspring resemblance, the resulting variation remaining open to natural selection while they are maintained. The cases of the inheritance of metabolic disorders described above, as well as the example of the non-genetic transmission of environmentally triggered responses (box 4), suggest much higher levels of transgenerational stability. Studies in *Caenorhabditis elegans* have shown the role of various types of non-coding RNAs in the inheritance of adaptive responses, with transgenerational effects spanning over more than 25 [78] and even 80 generations [79]. The latter example, transposed at the human timescale, would mean that environmental effects that occurred about 2000 years (taking 25 years per generation) ago and that might have affected the ancestors of currently living persons might still be affecting those person's phenotype today. However, more research is needed before extrapolating these results to long-lived species as the physical timescale of an epigenetic mark might also play a role. Obviously, despite the fact that this inheritance of variation would rest entirely on variation in non-genetic information, even the most demanding populational estimation of heritability would incorporate such variation into the estimate of heritability that is invariably claimed to be of genetic (i.e. DNA sequence) nature.

Similarly, in the example of animal cultural transmission (box 5), theoretical considerations [80] and experiments [66] led to conclude that local traditions might persist over hundreds if not thousands of generations. This would provide plenty of time for natural selection to fix those inclusively heritable variants and thus provide enough time for some form of genetic assimilation to occur [19,77,81].

### (c) The reversibility/fidelity trade-off

A common point of the above examples is that the inherited adaptation remains reversible. Although commonly viewed as a weakness of non-genetic inheritance, from an adaptive viewpoint this reversibility can on the contrary be seen as a strength, allowing parents to mould their offspring's phenotype to current conditions, while still allowing them to adopt another phenotype in case of further environmental change. To the contrary, while the strength of sequencic inheritance undoubtedly is in its stability, its weakness lies in its lack of reversibility making it inappropriate to allow adaptation to relatively fast-changing environmental characteristics. These considerations suggest that genetic and non-genetic inheritance systems constitute complementary mechanisms of adaptation to an environment whose many changes occur along very different timescales [3], a subject to which we now turn.

## 4. The timescales of accommodation and adaptation

### (a) Information stability of the various inheritance systems

**Figure 1.**
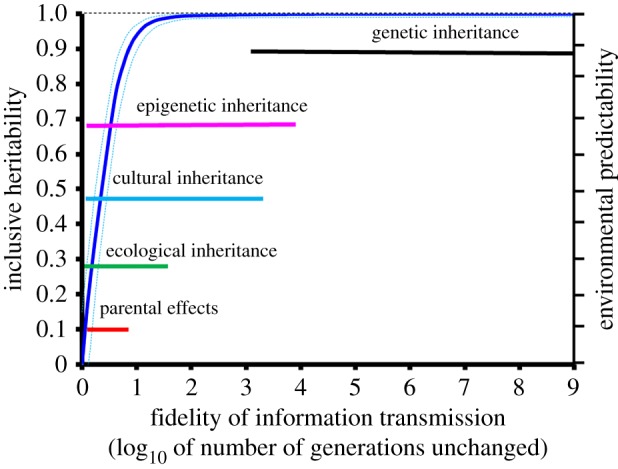
Relationship between transmission fidelity, environmental temporal predictability, and the maximum participation to inclusive heritability. x-axis: transmission fidelity (i.e. expected number of generations between successive changes). It can also be understood as the irreversibility of information [84,85]. Ranges of fidelity along the x-axis of the various inheritance-systems are indicated in colours. Left y-axis: maximum contribution to inclusive heritability. The blue solid curve represents the maximum heritability of an inheritance system having the fidelity indicated on the x-axis. Right y-axis represents the associated range of environmental predictability taken as a measure of the timescale of environmental variation. This range is represented by the vertical distance between the light blue lines for a given transmission fidelity along the x-axis. The area between the blue lines thus represents the vertical range of timescale of environmental predictability to which adaptation is possible with an inheritance system having fidelity *X*. This area appears narrow because the x-axis is in log_10_. Too unstable an inheritance system cannot encode adaptation to long-lasting environmental characteristics. Conversely, below that area, an inheritance system is too stable to adapt to fast-changing environmental characteristics. Thus, to enable adaptation, the fidelity of an inheritance system should more or less match the timescale of environmental change. (Online version in colour.)

The stability of genetic (i.e. sequencic) information is very high, with estimated probabilities of change per nucleotide and generation sometimes as low as 10^−9^ [82,83] (figure 1). Concerning transgenerational epigenetic inheritance, the stability of DNA methylation patterns across generations is only estimated for CpG pairs where it varies from 10^−2^ [86] to 10^−4^ per CpG pair per generation [83], orders of magnitude higher than for genetic change (figure 1). While changes in methylation status at single sites are relatively frequent [87], changes in methylation at scales similar to the ones distinguishing epialleles are much rarer and sometimes maybe in the same low range as those of some DNA mutations [84,87]. Stable transcriptional silencing by piRNAs has been described in germline cells in *C. elegans*, where histone configuration associated with longevity can last for at least 20 generations [88]. Estimates are lacking for other known epigenetic inheritance systems. The stability of parent–offspring resemblance by parental effects can be ranked lowest as changes are supposed to occur every few generations. Information stability can be higher for ecological inheritance (the transmission of modified selection pressures to the offspring, [89]) and cultural inheritance (the social transmission of behaviour, box 5, figure 1, [80]).

### (b) Information stability and the rate of environmental change

Differences in transmission fidelity suggest different evolutionary roles for the various inheritance systems [3,54,90–93]. In particular, different degrees of transmission fidelity can match different tempi and modes of environmental changes [3,5,84,85,93].

Environmental changes occurring within one generation (bottom and extreme left of figure 1) are unlikely to select for vertical transmission of information [85,93,94], but rather are expected to select for within generation information transfers (e.g. communication and non-transmitted phenotypic plasticity). Then, phenotypic plasticity leads to phenotypic accommodation (e.g. [95, p. 147]).

As soon as environmental characteristics remain stable over more than one generation (bottom or left part of figure 1), organisms able to transfer adaptive phenotypes across generations should be favoured as they can mould their offsprings' phenotypes to the prevailing conditions that are likely to be faced along their lifespan [85,91,93,94]. Parental effects, for instance, shape offspring phenotypes to conditions likely to persist over a few generations. This is the case when females transfer antibodies against current specific parasites [96,97] and stop doing so when the parasite disappears from the habitat [98]. Such parental effects are thus updated at every generation, allowing the tracking of environmental change in real time. They nonetheless contribute to parent–offspring resemblance, and thus to heredity. As they have strong fitness effects, they can strongly affect the fate of populations.

With environmental characteristics that persist over many generations (values ranging around 1 and 2 on the x-axis of figure 1), natural selection may have the time to change the encoding into more stable inheritance systems [19,77,84,90]. Even rarer changes should favour inheritance systems with ever increasing fidelity (moving towards the right on the x-axis of figure 1).

The maximum possible contribution to inclusive heritability [15,29] of the various inheritance systems increases with transmission fidelity (left y-axis and dark blue solid curve in figure 1). Thus, because of their persistence, the real contribution of epigenetic and cultural inheritance to inclusive heritability can be substantial, as demonstrated in plant and animal studies [80,99–102].

From an evolutionary point of view, epigenetic mechanisms also cast a new light on the evolution of multicellularity: it is possible that transgenerational epigenetic inheritance constitutes a by-product of mechanisms of cell differentiation and accommodation to varying environments at the intragenerational timescale, but it is also possible that epigenetic inheritance in unicellular organisms favoured the evolution of multicellularity by producing stable phenotypic variation [10,14,30,103]. For instance, increasing the amount of intrinsically disordered proteins and alternative splicing in ancestral unicellular organisms may have both ‘increase(d) protein functionalities without increasing proteome or genome size’ [104], an important prerequisite for the evolution of multicellularity. It appears that signalling molecules of multicellular organisms include many intrinsically disordered domains [104], the maintenance of which may have been facilitated by epigenetic inheritance.

Overall, these considerations show that the various components of inheritance play complementary roles in heredity, with selection favouring the fine tuning of the inheritance system to the regime of environmental variation [85,91,93]. Non-genetic and genetic inheritance thus occupy different ‘timescale niches’, each setting the stage for the other to become more specialized in its own timescale. Genes were thus probably able to evolve increasing fidelity up to the point of becoming ‘sanctuarized’ and remaining unchanged for millions of generations. Reciprocally, this makes genes particularly unsuitable to allow adaptation to fast changing environments, which is made possible by inheritance systems with lower transmission fidelity.

### (c) Information stability and cumulative evolution

Accumulation of variation requires stable information systems. However, short-lived information systems can also favour cumulative evolution. For instance, they can play the role of a temporary buffer to selective pressures allowing more stable variants to evolve [84,105,106], and it has long been shown that developmental adaptive processes can accelerate adaptation and evolution [107,108], and even short-lived variants can affect the genetic structure of a population if they have a strong effect on fitness [84], as is the case for instance with maternally transferred immunity. We also documented elsewhere what can be seen as switches among inheritance systems in a form of epigenetically-facilitated mutational assimilation ([19], and see [90]).

### (d) Transposing the Fourier transform to the study of inheritance

The multiscale nature of inheritance could thus be apprehended with an analogy. Inheritance, and more generally phenotypic variation, with their multiplicity of timescales ranging from millionths to millions of generations, may be thought of as functions of time analogous to soundwaves. Soundwaves (and other functions of time) can be analysed by performing a Fourier transform, an operation decomposing the sound signal into the frequencies (inverses of timescales) that make it up. Similarly, studying the temporal aspects of phenotypic variation (intra- and inter-generational) in all their components would require performing a sort of Fourier transform analysis of its various frequency components and integrating them into a single inheritance function of time.

## 5. Sources of phenotypic variation: when does early life start?

**Figure 2.**
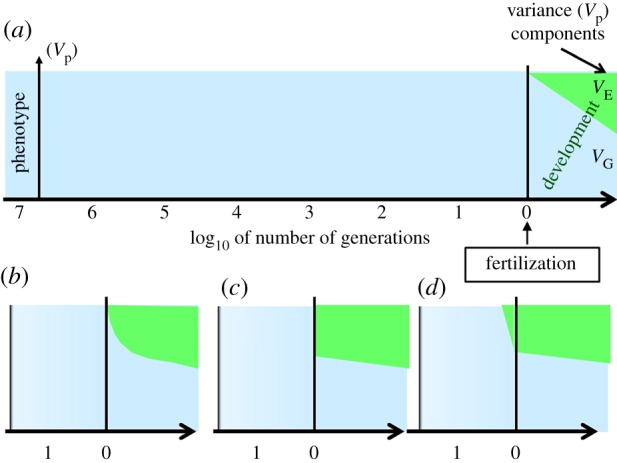
Sources of phenotypic variation according to neo-Darwinism. x-axis: log_10_ timescales of the number of generations before the fertilization of a given organism. The present time is at the far right end. (*a*) As formalized in the classic decomposition of phenotypic variation (*V*_P_ = *V*_G_ + *V*_E_), the role of *V*_G_ is illustrated by the light blue surface; that of *V*_E_ by the green surface. (*b*) and (*c*) represent various types of dynamics of environmental effects on the phenotype during development and (*d*) adds the effect of parental effects. (Online version in colour.)

In a classical neo-Darwinian view, an organism's phenotype results from the effects of its genetic information acquired at fertilization (figure 2, light blue areas), as well as of all environmental effects that influenced its development since fertilization (figure 2, green areas). The dynamics of environmental effects on development can take various forms. A straight line represents cases when environmental effects are equally effective on the phenotype during the whole lifetime (figure 2*a*). However, the line is more likely to be concave as early in life environmental effects are the most influential on the phenotype (figure 2*b*). Later in life, the slight negative slope of this bottom limit represents ageing. As early in life effects are far more influential than later in life, this green area can be drawn as a diamond shape (figure 2*c*).

In this perspective, parents do not transfer anything other than genes to their offspring, so that development only starts at fertilization (figure 2). A widely accepted exception is that of parental effects, when parents transfer to their offspring (often through the egg) hormones, antibodies or other molecules. This exception expands development over one (parental effects) or two (grand-parental effects) generations, and no more (figure 2*d*).

**Figure 3.**
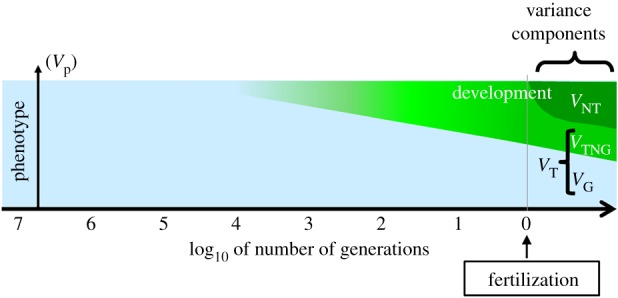
Sources of phenotypic variation according to the inclusive evolutionary synthesis. Legend as in figure 2. The wide triangle expanding thousands of generations in the past depicts the effect of non-genetically inherited adaptations to new environmental characteristics that ancestors acquired and transferred to their descendants. On its far left end, its colour blends into the light blue colour (representing genetic information) to illustrate the fact that at such long timescales non-genetic processes may become encoded into the DNA sequence in a form of epigenetically-facilitated mutational genetic assimilation [19]. Parent–offspring resemblance thus involves a variety of processes of transfer of information across generations all participating in heredity. Brief examples of the reality of inheritance of acquired adaptations are provided in the text and boxes 2–5. Conventions as in Danchin *et al.* [15]. *V*_NT_, non transmitted phenotypic variance; *V*_TNG_, transmitted non-genetic variance; *V*_T_, transmitted variation, whatever the mechanism of transmission; *V*_G_, genetic variance. (Online version in colour.)

Parental effects excepted, the claim that organisms may transmit some acquired traits across generations has been generally qualified as ‘Lamarckian’, a view which fell out of favour as neo-Darwinism gained power over the last century (reviewed in [90]). But from a purely Darwinian viewpoint this is paradoxical since recent ancestors experienced an environment that is a far better predictor of the offspring's life environment than its more ancient ancestors that lived thousands or millions of generations ago, and from which they inherited their genes. Thus, any organism becoming able to transfer recently acquired information about stable environmental characteristics to its offspring should be favoured by natural selection as its offspring would fare better than others that would only inherit genes (i.e. DNA sequences). This strongly suggests that organisms should have evolved ways of transferring recently acquired information about current environment to their offspring in a possibly relatively persistent manner. Such intergenerational transfers of accommodations to recent past environments are likely to significantly influence the way genes are expressed in interaction with the current environment in descendant organisms (i.e. a process that closely corresponds to what we call development, see glossary) for as long as these transfers persist. Thus, development (i.e. a pattern of gene expression, figure 2, green area) in effect initiated well before the fertilization that gave rise to a given organism (figure 3). This conclusion fits with the claim that segments of life corresponding to biological individuals are not necessarily delimited by unique and sharp events such as fertilization [109]. As a consequence, studies of developmental, ecological and evolutionary processes are along a continuum rather than on opposite sides of an artificial border [110,111].

## 6. Rejuvenating Darwinism from neo-Darwinism

### (a) Small non-coding RNAs as major but largely overlooked inheritance molecules

While the second half of the twentieth century hyped the role of the DNA molecule, the RNA molecule is now confirmed as being another major molecule of inheritance [19,60], indeed encoding information over shorter terms than DNA, but still with effects spanning over many generations. In effect, small non-coding RNAs (sncRNAs) can be produced in various somatic cells and then released and systematically distributed in the body organs ([18], reviews in [60,112–115]) where they can affect gene expression [78]. In a sense, sncRNAs are hormones, and are perfect candidates to mediate environmental effects as they can be produced in tissues that sense the environment (i.e. neurons and the brain for instance), and move to different body parts to affect gene expression [18]. When these modifications affect the germline, they can persist for many generations ([78,79], review in [115,116]).

**Figure 4.**
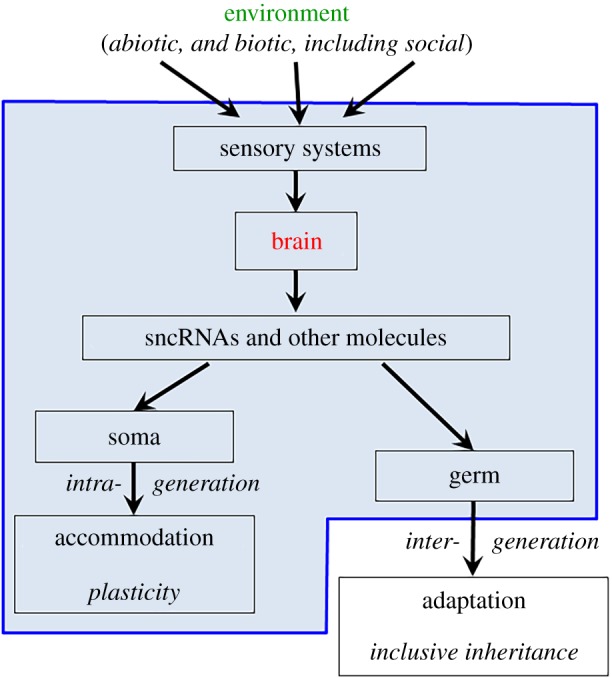
A potential pathway by which environmental effects may become inclusively heritable. We illustrate the case of brained organisms. In non-brained organisms the pathway may be similar, with other somatic or cellular (in unicellular organisms) structures playing the cognitive role of the brain. The blue box encloses all processes occurring within a single individual. (Online version in colour.)

These properties of sncRNAS lead us to now propose a pathway by which the environment may generate inherited adaptations (figure 4). First, environmental factors are detected by an organism's senses. In all organisms, those senses are based on calcium pumps at the cellular level. In plants, this sensitivity to environmental factors is well documented, both in relation to abiotic and biotic factors, for instance at the root level. In animals, senses may exist in many tissues in the organism and most often involve neurons. The resulting information is then processed by the organism. This processing can take many different forms. In organisms with brains (figure 4), this processing probably mainly involves the brain, which plays a central role in information processing, but in organisms without brains, including plants and microorganisms, other somatic structures play this role. These anatomical structures produce specific molecules (probably mainly sncRNAs) that circulate and modify gene expression in the whole body including somatic and germline cells (figure 4). In somatic cells, this can lead to phenotypic accommodation to environmental change. In the germline cells, this might generate epigenetic modifications that may affect the phenotype and persist for many generations, hence contributing to inheritance. Thus, RNA emerges as a major, and overlooked, inheritance molecule, complementing and interacting with the DNA.

### (b) Small non-coding RNAs as heirs of Darwin's gemmules?

Darwin's ‘pangenesis hypothesis’ speculated about the existence of ‘gemmules’ (germs of cells) supposed to originate in all parts of the body and move to the germline, allowing the inheritance of those parts' characteristics [117, p. 374]. Circulating sncRNAs provide one hypothesized function of gemmules. It has sometimes been claimed that even though Darwin's evolutionism included the use and disuse of organs as a cause of evolution, it was subordinated to natural selection as an evolutionary force [118, p. 274]. Later neo-Darwinians would drop this Lamarckian vestige from the theory, as a purely superfluous belief that Darwin held like all his contemporaries [31,119]. However, the theory of gemmules and its recent echoes in the findings regarding circulating RNAs indicate that a possible unity between natural selection and Lamarckian mechanisms was foreshadowed in Darwin's own theory of heredity.

### (c) The evolution of inheritance systems

In a 1932 lecture, Fisher argued that besides ‘the genetics of evolution’, there also existed an ‘evolutionary science of genetics’ [120], considering how the features of genes themselves (e.g. dominance, pleiotropy, hyper-dominance, mutation rates, heterosis, etc.) are shaped by evolution. Remembering that for Fisher genetics meant anything that is inherited (pre-DNA conception), we should undertake this research programme to study the evolution of inheritance systems, and accept that the pluralistic structure of inheritance is a result of evolution, and that it does not challenge a Darwinian view. We need to study the phylogeny of inheritance systems, exploring when and where specific mechanisms of inheritance originated, and how they were later recruited by exaptation.

For instance, we would expect species facing more variable environments to rely more on non-genetic inheritance systems [14]. A first contrast concerns plants and animals. There are indications that epigenetic inheritance is more pervasive in plants than in animals (at least in mammals).

‘The extent of methylation reprogramming in primordial germ cells is substantial and this limits the potential in mammals for epigenetic transgenerational inheritance. By contrast in plants where epigenetic reprogramming may not occur to such an extent in the germ line, examples of stable inheritance of epialleles over multiple generations are more common’ [121, p. 627].

More evidence for this claim is necessary, but a putative explanation might be in the difference in movement capacities between plants and animals. Being sessile with most of their seeds dispersing very short distances, plants can only adapt on the spot, thus forcing them to rely more on inheritance systems with fast turnover [122]. On the other hand, animals able to move can quit degrading habitats to establish in currently more suitable ones, thus buffering environmental change. Animals (and particularly migrating animals such as birds or whales) should thus be less prone to using inheritance systems with high turnovers and could be expected to rely relatively more on sequencic than non-sequencic inheritance [14]. We need more exploration of the links between species movement abilities and the relative role of genetic and epigenetic components, for which methods are available [123,124].

### (d) A historical parallel

In this paper, we proposed to articulate neo-Lamarckism and neo-Darwinism around the idea that inclusive inheritance systems are complex adaptations to environments varying in complex ways [90]. A historical parallel can be drawn between the emergence of this inclusive evolutionary synthesis and the emergence of another theoretical framework in evolutionary biology, that of the neutral theory of molecular evolution.

The neutral theory of evolution originated to accommodate the discovery that proteins were much more polymorphic than expected [125], a fact revealed by the then novel techniques of protein electrophoresis. This theory emphasized the role of drift in forging the nucleotidic composition of the genomes through evolutionary time, which potentially explained this unexpected variation [126]. While neutral evolution was first seen as antagonistic to Darwinian mutation–selection processes [127], these two visions were later recognized as compatible, with fitness effects of mutations ranging on a continuum from the neutral to the all-or-nothing [128]. Kimura himself did not view neutralism as denying selection, and thought that the inflation of neutral alleles could even result from natural selection. Today, neutral models constitute an excellent null model for testing selection effects [129]. Furthermore, there is a potential benefit in accumulating neutral mutations, as these may constitute a reservoir of variation in case of environmental change.

The recent emphasis on non-genetic inheritance presents us with an analogous history. New technologies have highlighted the existence of real discrepancies between expectations and observation. For instance, we were stunned by the discovery that there are many fewer coding genes than expected (19 000–30 000 in mammalian species). Additionally, we discovered that genetic (sequencic) variation is not sufficient to explain the complexity of heritable disorders that were considered as ‘genetic disorders’ on the sole observation that they were statistically transmitted (see the case of the missing heritability, [4,5,130]). Similarly to the initial vision of neutral variation, non-genetic inheritance is often considered to go against neo-Darwinism. However, one can generalize evolution (e.g. by natural selection) as a process acting on *any variation that is inclusively heritable*, be it genetic or non-genetic, and that determines either a trait, or the transmission of a trait [8,111]. Thus conceived, the emerging inclusive evolutionary synthesis constitutes a broadening of the Modern Synthesis, rejuvenating the original Darwinian viewpoint [19].

## 7. Early in life effects: when Darwin meets Lamarck

**Figure 5.**
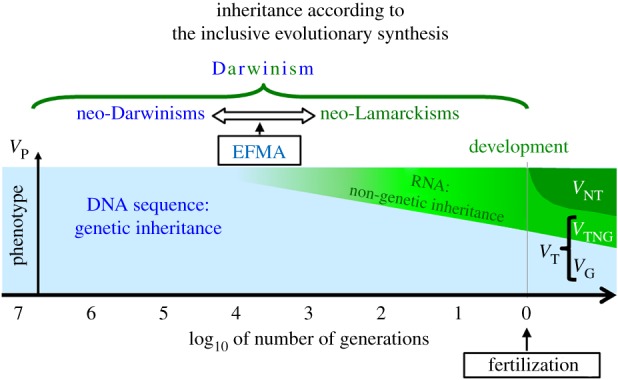
The concept of inheritance in the inclusive evolutionary synthesis. This synthesis unifies neo-Darwinism with neo-Lamarckism by rejuvenating Darwin's vision of inheritance. Blue hue (to the left) depicts the role of genetics, which constitutes the core idea of neo-Darwinism, stressing the major role of the DNA sequence. Green hue (to the right) represents components that are inherited non-genetically and are in part under environmental influence, which constitutes the main claim of (neo)-Lamarckism. RNAs constitute one of the major molecules of non-genetic inheritance of recently acquired characters. EFMA: Epigenetically-Facilitated Mutational Assimilation [19]. (Online version in colour.)

Defenders of the original Modern Synthesis acknowledge the existence of non-genetic inheritance, but object that epigenetic inheritance is too labile to play an evolutionary role. This corresponds to the common claim that epigenetics and, more generally, ‘soft inheritance’ pertain to proximate causes, and therefore do not really justify modifying evolutionary theory [131]. A common answer to this claim is that even if epigenetically transmitted changes lasted only a few generations (note, however, that evidence for their persistence is accruing [5,15,19,84,90]), they may nevertheless strongly affect phenotypic variation and fitness and therefore inflect the course of evolution by natural selection [111]. Another, more long term, kind of evolutionary impact is that epigenetic inheritance may affect the stability of the genetic system [19]. Epigenetic marks are considered mutagenic under certain circumstances [132,133], implying that they can affect the stability of physically linked more stable variants (the DNA sequence). They may thus play the role of a hub towards more stable information encoding in a form of Epigenetically-Facilitated Mutational Assimilation (EMFA, figure 5, [19]), somehow enabling the genetic and non-genetic systems to ‘talk’ to each other [8,19,77,90]. To sum up, although a common objection against epigenetic inheritance in evolutionary biology is that its effect are too labile as compared to the effects of genes, here we claim that it is precisely *because* epigenetic inheritance is shorter lived that it can be evolutionarily relevant, in the way explained here and in Danchin *et al.* [19].

Non-genetic inheritance invites us to consider that early in life effects, and more generally development, initiate many generations before fertilization (figure 5, green area). A consequence is that the boundary we often draw between development and inheritance (or between the individual organism and lineages) depends on the timescale of study [111]. This vision of inheritance unifies the supposedly incompatible neo-Darwinian and neo-Lamarckian views along a continuum of timescales (figure 5). In the very long term, evolution by natural selection may rely on genetic information, which is highly preserved from environmental changes, and prepares the offspring for highly stable environmental characteristics. At shorter timescales, heredity results from a variety of non-genetic inheritance systems. In this viewpoint, the resetting of some methylation marks at meiosis makes perfect sense regarding the marks that are supposed to target environmental features with relatively high rates of change. Nonetheless, it is now becoming clear that some epigenetic marks escape such reprogramming, giving them a role in adaptation at other timescales, and thus participating to a more inclusive concept of early in life effects.

## Data accessibility

This article has no additional data.
